# Building tobacco control research in Thailand: meeting the need for innovative change in Asia

**DOI:** 10.1186/1478-4505-10-3

**Published:** 2012-01-28

**Authors:** Stephen L Hamann, Jeremiah Mock, Sibasis Hense, Naowarut Charoenca, Nipapun Kungskulniti

**Affiliations:** 1 Tobacco Control Research and Knowledge Management Center, Bangkok, Thailand; 2 Osaka University, Center for the Study of Communication-Design, Osaka, Japan; 3 Apollo Hospitals Educational and Research Foundation, Hyderabad, India; 4 Faculty of Public Health, Mahidol University, Bangkok, Thailand

**Keywords:** tobacco control, smoking, policy, research, capacity building, secondhand smoke, Thailand, Asia

## Abstract

**Introduction:**

In low- and middle-income countries (LMICs) over the past two decades locally relevant tobacco control research has been scant. Experience shows that tobacco control measures should be based on sound research findings to ensure that measures are appropriate for local conditions and that they are likely to have an impact. Research should also be integrated within tobacco control measures to ensure ongoing learning and the production of knowledge. Thailand, a middle-income country, has a public health community with a record of successful tobacco control and a longstanding commitment to research. Thailand's comprehensive approach includes taxation; bans on tobacco advertising, sponsorship and promotion; smoke-free areas; graphic cigarette pack warnings; social marketing campaigns; cessation counseling; and an established tobacco control research program. The purpose of this study was to document and analyze the development of tobacco control research capacity in Thailand and the impact of research on Thai tobacco control measures.

**Method:**

We used mixed methods including review of historical documentation and policy reports, qualitative interviews with key members of Thailand's tobacco control community, and an analysis of research productivity.

**Findings:**

In Thailand, tobacco control research has evolved through three phases: (1) discovery of the value of research in the policymaking arena, (2) development of a structure to support research capacity building through international collaborations supported by foreign funding agencies, and (3) delivery of locally relevant research made possible largely through substantial stable funding from a domestic health promotion foundation. Over two decades, Thai tobacco control advocates have constructed five steppingstones to success: (1) adapting foreign research to inform policymaking and lobbying for more support for domestic research; (2) attracting foreign funding agencies to support small-scale research and capacity building; (3) participating in multi-country research and capacity building programs; (4) using collaborative experiences to demonstrate the need for domestic support of locally relevant research; and (5) maintaining an unwavering commitment to research while being vigilant to ensure continued research support.

**Conclusion:**

The evolution of tobacco control research in Thailand provides examples of steppingstones that LMICs may be able to use to construct their own tobacco control research pathways.

## Introduction

Tobacco control is a process of undertaking measures to reduce tobacco use and eliminate exposure to tobacco and smoke. Experience shows that tobacco control measures are much more likely to be effective when they are based on solid, locally relevant public health research [1]. This is the case because to be effective, tobacco control measures have to be designed to address local problems and be appropriate for local conditions.

In low- and middle-income countries (LMICs), over the past two decades very few people have been working full-time on tobacco control. Even fewer have been producing tobacco control research that is relevant for their populations [2]. Over the past two decades, some LMICs have achieved successes in implementing tobacco control measures despite the lack of locally relevant research. Nevertheless, many more measures have resulted in untold failures. Still more have been implemented without a research component, thus resulting in missed opportunities to learn lessons from experience and generate knowledge.

In most LMICs, the tendency, in the absence of locally relevant research, has been to "copy and paste" a tobacco control measure that was undertaken in another country, particularly a wealthy country that produces a lot of tobacco control research. This approach, albeit pragmatic and sometimes workable, has been fraught with problems because sociocultural and political-economic contexts vary substantially from country to country, and even within countries.

In LMICs, the lack of tobacco control research is a serious impediment to reducing tobacco use and eliminating exposure to tobacco and smoke. The large majority of the world's tobacco users live in LMICs. Extensive biomedical evidence shows that tobacco use and secondhand smoke exposure cause a wide range of non-communicable diseases (e.g., heart disease, cerebrovascular disease, lung cancer) and premature death [3]. Many LMICs are experiencing an epidemiological transition in which the prevalence and incidence rates of traditionally widespread communicable diseases (e.g., measles, polio, dysentery) are declining while the prevalence and incidence rates of non-communicable diseases are rising [4]. Amidst these conditions, transnational tobacco companies (TTCs) are targeting consumers in LMICs aggressively since LMICs are their only markets with major growth potential [5].

Clearly, the fact that national governments in nearly all LMICs have become signatories to the World Health Organization's (WHO) Framework Convention on Tobacco Control (FCTC) shows that these governments recognize that tobacco use is a serious problem in their countries. However, the amount of tobacco control research LMIC national governments support is not in any way proportional to the magnitude of the problem [6]. International philanthropic efforts have filled this funding gap partially, but in almost all LMICs tobacco control research continues to be scant and inadequate.

People in LMICs undertaking tobacco control measures usually have no local research findings from which to demonstrate the potential effectiveness of a "copy-and-pasted" measure. A still greater problem is that typically there are almost no local research findings that people can use to develop their own indigenous measures they design from the outset to address the conditions in their own specific cultural and political contexts. The lack of research in LMICs presents an even greater problem. Experience shows that research should be an integral part of the design and implementation of tobacco control measures so that those who implement a measure can learn and adjust throughout the implementation process while generating knowledge that can provide an evidence base for developing future measures.

Some observers have suggested that the barriers to tobacco control research in LMICs include lack of data standardization, weak communications networks, inadequate human and material capacity to conduct research, and lack of funding [7]. Another barrier may be that national governments tend to allocate scarce resources to address immediate pressing health problems rather than funding activities, including research, to prevent future problems.

Tobacco control advocates in Thailand have faced all of these circumstances. To a considerable degree, they have worked through or around them. The evolution of tobacco control research in Thailand provides examples of steppingstones that people in LMICs may be able to use to construct their own tobacco control research pathways. In this paper, we report on why research has been an important component of Thailand's comprehensive approach to tobacco control. We describe how Thailand's tobacco control research capacity has developed over time. We also show why research has been important to ensuring successful tobacco control in Thailand. These findings may be valuable for consideration in LMICs, particularly in Asia.

### Tobacco use situation in Asia

If evidence-based preventive measures are not undertaken globally on a widespread scale soon, the number of tobacco users worldwide could increase from 1.4 to 1.9 billion by 2030 [7]. In this scenario, about 80% of the projected mortality from tobacco-related diseases will occur in LMICs, the majority in Asia. Tobacco use rates and secondhand smoke exposure rates are high and/or increasing in most Asian countries. China and India alone have large populations of tobacco users who will suffer from tobacco-related diseases and premature death. South and Southeast Asia have one third of the world's smokers and more than 1.4 million die each year from tobacco-related deaths [8].

### Comprehensive approach to tobacco control in Thailand

Thailand is a middle-income country in Southeast Asia that has received attention for having a tobacco control community with a record of implementing successful tobacco control measures. The smoking prevalence rate in Thailand has fallen steadily from 59.3% for males and 5.0% for females in 1991 to 40.5% and 2.0% in 2009 respectively, an aggregate 35% drop over 18 years [9]. While many countries rely heavily on tax policy to reduce tobacco use, Thailand's public health community has used a comprehensive approach that, in addition to taxation, includes bans on tobacco advertising, sponsorship and promotion; laws mandating smoke-free indoor and outdoor areas; graphic cigarette pack warnings; social marketing campaigns to educate and encourage smoking cessation; a national smoking cessation telephone counseling warmline; and an established tobacco control research program. Accordingly, Thailand is one of only a handful of countries that has achieved nearly complete compliance with the World Health Organization MPOWER indicators for implementation of FCTC [10].

## Methods

We used mixed methods to document and analyze the development of Thailand's tobacco control research capacity and the impact of research on Thai tobacco control measures. We reviewed and synthesized information in historical documentation and from available policy development reports [11-14]. Additionally, we conducted and reviewed nearly 40 qualitative interviews with key members of Thailand's tobacco control community to understand their perspectives about structural changes in Thailand's tobacco control research capacity. Concurrently, we examined an existing analysis of Thailand's research productivity and we conducted a new analysis using bibliometric methods [15]. For our analytical strategy, we triangulated evidence generated through this mix of methods to formulate a holistic assessment of the changes in the tobacco control research structure in Thailand and the processes by which research has influenced Thai tobacco control efforts. This study benefits from an additional factor: All but one of the authors have experienced first-hand the structural changes and processes we describe herein [16,17].

## Findings

In Thailand, tobacco control research has evolved through several phases that are somewhat like the main phases of research itself: discovery, development and delivery. When tobacco control started in Thailand more than two decades ago, it was necessary for the few people working to reduce tobacco use to discover, together with the Thai government, that research was important. Over time, tobacco control activists and government officials began developing a research structure while building Thai research capacity. As these efforts took hold, it became possible for Thai researchers to deliver research products that were relevant to issues in the Thai context. We describe this progression from discovery to development to delivery below.

### Evolution of the role of research in the Thai tobacco control model

In the late 1980s - the early period of tobacco control in Thailand - Richard Peto, a famous tobacco epidemiologist, urged a small group of Thai tobacco control advocates to undertake several tobacco control studies. However, at that time, there were very few tobacco control resources in Thailand. Moreover, everyone's attention was focused on the tobacco trade dispute between the Thai government and the US Trade Representative's Office over American tobacco companies' desire to export to the Thai market. Although the Thai government was unsuccessful in restricting foreign tobacco companies from selling in the Thai market, the Thai government pushed ahead with two comprehensive tobacco control laws: one to control tobacco products and the other for the protection of non-smokers from exposure to tobacco smoke. Lawmakers wrote these laws based on advice from Thai tobacco control advocates who had studied research evidence from other countries.

In the late 1980s, three Thai tobacco control NGOs began gaining strength and legitimacy. Together, they succeeded in shepherding a "tax for health policy" law through parliament in 1993 to increase tobacco excise taxes pegged to the cost-of-living. These Thai tobacco control advocates were able to use international research to make projections of the number of Thai children who would be spared from becoming smokers and dying prematurely. Advocates also used international research to show policymakers the beneficial economic effects of a tobacco tax policy. At this time, Prakit Vateesatokit, one of Thailand's leading tobacco control advocates, mused about the lack of research evidence in Thailand saying, "Research is still needed, but if action is delayed by demands for country-specific proof, many countries may never be able to make speedy progress in tobacco control. It is not uncommon for some politicians to request evidence from research as a pretext to block or delay tobacco control measures" [18].

Throughout the 1990s, Thai tobacco control advocates became more adept at leveraging available international research findings to achieve policy change. At the same time, an important transformation was taking place in Thailand about the use of structural power. In this period, Thailand's civil society organizations increased rapidly in number and size, and they pushed fervently for political representation and moral change [19]. By 2000, there were over 500 Thai non-governmental organizations (NGOs) conducting health-related activities. Tobacco control NGOs were a leading part of this civil society upsurge, and they established the model for pulling together pieces of research to influence government policymaking effectively [20].

In the mid-1990s, Thai tobacco control NGOs began a campaign to develop Thailand's domestic tobacco control research capability. Although the Tobacco Consumption Control Office (TCCO) in the Ministry of Public Health gave some small grants for research, TCCO commissioned most of the studies they funded [21]. During this period, the only other tobacco control entity focusing on research in Thailand was the Tobacco Control Policy Research Network (TCPRN), an NGO that connected researchers interested in conducting studies in Thailand and the region. In 1995, TCPRN received the endorsement of the Asia-Pacific Association for the Control of Tobacco (APACT) [22]. TCPRN conducted activities until 2000 when a new era of tobacco control research began to emerge.

In the 1990s, at the same time that Thai tobacco control advocates were working to establish greater domestic tobacco control research capacity, international agencies were beginning to recognize and support tobacco control activities in Southeast Asia. For example, Canada's International Development Research Centre, through its Research for International Tobacco Control program, worked with the Thailand Health Promotion Institute to forge a Southeast Asia agenda for tobacco control.

In November 1998, a small group of tobacco control researchers participated in an Asian regional tobacco control meeting in Pattaya, Thailand with the ambitious goal of drafting a multi-disciplinary regional research agenda. A subsequent article in Tobacco Control reported on four main research needs that participants at this consultative meeting identified: (1) a lack of standardized and comparable data, (2) the absence of a network for research communications, (3) a lack of adequate research capacity, especially regarding economic and policy analysis, and (4) the need for mobilization of human and financial resources [23].

Beginning in 2000, Thailand attracted the support of several foreign organizations interested in tobacco control research in LMICs, notably the Rockefeller Foundation, the Institute for Global Tobacco Control's Global Tobacco Research Network (GTRN) based at the Johns Hopkins University Bloomberg School of Public Health, the International Tobacco Evidence Network (ITEN), and the United Nations Foundation [24]. The Rockefeller Foundation funded GTRN to conduct research capacity-building activities and coordinate multi-country studies through the newly established Southeast Asia Tobacco Control Alliance (SEATCA). Up to 2004, Rockefeller Foundation's "Trading Tobacco for Health" program supported small research projects in Thailand and in other SEATCA member countries [25]. Some Thai researchers began conducting local studies to highlight the need for policymakers to support regulatory improvements. For example, several foreign organizations supported a series of small studies examining secondhand smoke exposure levels in restaurants, homes, pubs and bars [26-30]. These studies documented the consequences of secondhand smoke exposure for nonsmoking workers, patrons, mothers and children. For more than a decade, these studies have provided advocates with evidence to lobby policymakers to strengthen Thailand's Nonsmokers' Health Protection Act [31].

In the early 2000s, despite more than a decade of tobacco control efforts in Thailand, international and domestic studies showed a continued lack of public awareness about tobacco control and a substantial need to better inform the public about the hazards of tobacco use and secondhand smoke [12,23]. One effort attempted to enlist Thai health professionals in greater advocacy-related research by demonstrating how health professionals in other countries had influenced policy development and implementation. This effort met with little success since limited structural support or resources could be offered to health professionals who were encouraged to participate. Nevertheless, in this period, health professionals inside and outside government began to assert structural influence. The Thai Health Systems Research Institute (HSRI) advocated for the establishment of innovative organizations to advance tobacco control and health promotion. In a time when few financial resources were available to support tobacco control research, HSRI and a few NGOs initiated research projects that clearly had the potential to promote health [32].

### ThaiHealth Foundation: a new era of research infrastructure and funding

Since the mid 1990s, Thai tobacco control advocates had felt that for research to be relevant to the Thai context, it should be conducted in Thailand at the local and national levels, and thus, it should be funded from within Thailand at those levels [1]. So, after many years of planning and political work, in 2001 Thai tobacco control advocates succeeded in persuading lawmakers to pass a law to establish the Thai Health Promotion Foundation (ThaiHealth). ThaiHealth was initially modeled on VicHealth in Victoria, Australia along with several other national health promotion foundations. Revenue for ThaiHealth was established from a new 2% earmarked tax on tobacco and alcohol importers and manufacturers. Although ThaiHealth supports a wide range of health promotion activities, a central focus is to build up a cadre of committed tobacco control researchers. Over the past decade, ThaiHealth has been successful building human research capacity because of its sustainable stream of funding and established mechanisms for supporting Thai academics' programs of research.

Since the Thai Government does not provide many resources for tobacco control research, the addition of a funding mechanism from ThaiHealth has been an important resource. ThaiHealth has supported several tobacco research grant-making organizations, including the Tobacco Control Research and Knowledge Management Center (TRC) at Mahidol University, established in 2005 [33]. TRC has supported over 100 research projects in Thailand including at least 27 graduate student theses dealing with priority issues in Thai tobacco control [34].

In 2005, ThaiHealth supported the formation of the Thai Health Professionals Against Tobacco (THPAT) and Teachers Against Tobacco Network (TATN). THPAT includes doctors, nurses, dentists, pharmacists, public health and other health professionals while the TATN includes primary and secondary school teachers throughout the country [35]. This support has produced greater involvement of health professionals and teachers in tobacco control research as evidenced by their increased participation in tobacco control conferences regionally and internationally. For example, 95 Thais participated in the last APACT Conference in Sydney, Australia in 2010, the largest contingent coming from outside Australia.

While ThaiHealth has become the primary funder of tobacco control research in Thailand, some Thai researchers have continued to carry out research activities through collaborations and funding from foreign sources. These have included the Rockefeller Foundation, American Cancer Society, Johns Hopkins University Bloomberg School of Public Health, the International Tobacco Control Policy Evaluation Study (ITC), Roswell Park Cancer Institute's Transdisciplinary Tobacco Use Research Center, Flight Attendant Medical Research Institute, and Bloomberg Philanthropies.

### Increased research productivity

In 2006, TRC published a review of the amount and type of tobacco-related research conducted in Thailand in the period 1976-2006 [15]. Overall, this review identified 325 studies, with 88% conducted after 1991 when the government began limited funding of research through the TCCO and in ThaiHealth's initial period of funding research. The review showed that the number of Thai tobacco control studies increased over time, but a majority of the studies identified (53% of the total) were student theses, often unpublished. While this review produced a valuable assessment of research productivity, it was not totally comprehensive because it only included research catalogued in Thai universities on limited databases with narrow inclusion and exclusion criteria. Also, the review only encompassed the preliminary 5 years of ThaiHealth's grant making activities. So the study could not detect the full effect of ThaiHealth's research support program.

To measure the effects of ThaiHealth's tobacco research support over a decade, we conducted a comprehensive bibliometric assessment of tobacco control studies conducted in Thailand and published in biomedical and social science journals. We searched the Scopus database using a search protocol modeled on a recent study of research productivity in New Zealand [36]. We examined the number of published tobacco research papers, the number of journals publishing Thai tobacco control research, and the number of Thailand-based first authors in the 10 years before ThaiHealth was established (1990-2000) and the ten years after (2001-2011). Our analysis showed 74 papers published in the 10 years before ThaiHealth was established and 376 published papers in the 10 years during which ThaiHealth funded tobacco control research. This represents a five-fold increase in the number of publications. In addition, there was also a five-fold increase in the number of Thailand-based first authors, and a 4.6 times increase in different journals publishing Thai tobacco-related research.

### Implications for increasing tobacco control in LMICs

Our analysis shows that in Thailand the structure and process of supporting tobacco control research has evolved through phases of discovery, development and delivery as the commitment to producing and using research to inform tobacco control measures has increased. This commitment is now reflected in ThaiHealth's sustained support of organizations delivering increased research productivity [33]. Thai tobacco control NGOs' increasing political influence has been both responsible for, and due to ThaiHealth's performance. Most importantly, today Thailand has an innovative structure for supporting tobacco control research because about a dozen Thai tobacco control advocates recognized that systems thinking about structures was needed to foster Thai control and local participation in research [37].

WHO and the GTRN have conducted studies assessing factors that foster and impede tobacco control research in LMICs. These studies have shown consistently that funding, infrastructure, resources and TTC influence are the most challenging impediments to building up tobacco control research [14,38]. The Thai tobacco control community has been successful in establishing substantial stable funding, building up infrastructure, and creating resources to support tobacco control research. They are still working to eliminate TTC influence. WHO has highlighted the ThaiHealth model of earmarked taxes to support tobacco control intervention programs. Our analysis shows that it is also important to recognize that a dedicated tax can establish substantial stable domestic funding for tobacco control research [39].

A recent analysis of trends in global development assistance shows that Southeast Asia has received special attention in terms of development assistance for tobacco control [40]. Undoubtedly, development assistance has contributed to Thailand's success in building up tobacco control research. But as the cliché goes, where there's smoke, there's fire. Our assessment shows that most of the development assistance came after the Thai tobacco control community had already started using research and campaigning for domestic research support, and long after domestic mechanisms for supporting tobacco control had been established. Thai tobacco control advocates' ongoing commitment to research is the "fire" that attracted international agencies to support tobacco control research in Thailand. The Rockefeller Foundation, for example, decided to establish the coordinating office of SEATCA in Thailand to foster capacity building and research delivery in Southeast Asia. Our analysis shows that even though tobacco control was established in other SEA countries, Rockefeller Foundation chose to base its program in Thailand because Thai NGOs had already demonstrated their commitment to research. This commitment to research was bolstered by foreign development assistance, but ironically the Thai NGOs experience of receiving foreign development assistance actually increased their desire to establish a substantial stable domestic source of funding. Through their experience in SEATCA, Thai tobacco control advocates realized it would be better to have Thais in control of the research agenda and funding decisions. Thus, they pushed the government even harder to establish ThaiHealth including a mandate, in addition to supporting health promotion activities, to provide a stable source of support for tobacco control research.

For LMICs, particularly those in Asia, Thailand's model and experience lays out five potentially useful steppingstones for building a national tobacco control research base:

1. In circumstances where domestic or foreign resources of support for locally relevant tobacco control research are limited, tobacco control advocates may be able to use research from other countries (e.g., epidemiological projections, economic projections about the impact of tobacco tax policy) to the degree that the foreign research can be adapted to the local circumstances to inform policymaking. Such "copy-and-paste" strategies should be used cautiously. Tobacco control advocates may also be able to use foreign research findings strategically to sensitize policymakers about the importance of research, thus laying a foundation for campaigning for domestic research funding.

2. As an intermediate step to building domestic research capacity, it may be necessary to attract foreign funding to support small-scale research and capacity building. When tobacco control advocates can demonstrate that they have used foreign research strategically and that they are committed to using domestic research, they will probably be more successful at attracting foreign funding.

3. Participating in foreign-sponsored multi-country research and capacity building programs can be a valuable way to build up some parts of a domestic research infrastructure. Through such opportunities, it is possible to have foreign experts assess the gaps and weakness in existing domestic research capacity. Tobacco control advocates can use such assessments to persuade policymakers to invest in new domestic research capacity to generate locally relevant evidence for policy and practice.

4. While it is important to be engaged in international collaborative tobacco control research, development assistance is seldom sustainable enough to ensure that a country can build up a stable research base over time. Moreover, while international collaborations allow a country's researchers to become part of international networks, much of the control of such collaborations almost invariably remains in the hands of foreign investigators and funding agencies. Tobacco control advocates can use such experiences with international collaborative research to prove to policymakers that their government needs to make strategic structural changes to create opportunities for domestic researchers to build their own country's path of tobacco control research.

5. In LMICs, there are always pressing problems, competing interests, and policymakers who do not see the value of research. Thus, tobacco control advocates must maintain an unwavering commitment to research to ensure sufficient stable funding. Also, they must be ever vigilant about attempts launched by domestic constituencies as well as TTCs to divert funds away from research.

## Conclusion

We are now in an era where tobacco control advocates and policymakers in LMICs generally recognize the importance of tobacco control research [41]. Still, in most LMICs, many steps lay ahead to move from recognition to building a robust structure and process for increasing research capacity. In Thailand, tobacco control advocates learned through challenging circumstances that research can be a vital instrument for policymaking [18]. The path that started with this discovery has not been easy. It has been a hard fought struggle to build a domestic research base that only now is producing locally relevant research based on local participatory assessments of domestic research needs. Finally, after more than two decades of sustained commitment to building up research capacity, Thai tobacco control advocates can drive the research agenda in pursuit of domestic tobacco control objectives. The path Thai tobacco control advocates have constructed may not be relevant for tobacco control advocates in some LMICs. Those whose circumstances are similar to the circumstances Thai tobacco control advocates have faced may be able to fashion similar steppingstones leading to discovery, development and delivery of research. At a minimum, the Thai model can provide inspiration for maintaining a commitment to research so as to ensure that every tobacco control measure undertaken can generate knowledge and learning, while having the greatest likelihood of reducing tobacco use and exposure.

## Competing interests

The authors declare that they have no competing interests.

## Authors' contributions

The study was conceived by SLH, designed by SLH and NC, undertaken by SLH, NC, NK, and SH, analyzed and written by SLH and JM. All authors read and approved the final manuscript.
